# Kinetic Analysis of Korean Traditional Dance Movements by Using Ground Reaction Force

**Published:** 2018-10

**Authors:** Kyeong-Won JUNG, Dai-Hyuk CHOI, Wi-Young SO

**Affiliations:** 1. Dept. of Korean Dance, College of Arts and Physical Education, Sejong University, Seoul, Korea; 2. Dept. of Physical Education, Graduate School of Education, Sogang University, Seoul, Korea; 3. Sports and Health Care Major, Korea National University of Transportation, Chungju-si, Korea

## Dear Editor-in-Chief

The art of dancing is conveyed through various combinations of body movements. Korean dance represents a unique challenge as this creative art form combines rapid tempo and large movement that commonly exceed the limits of stability of the body, placing a high demand on the structures of the musculoskeletal system which can result in injury (1–3). Among the various movements of Korean dance, three require skill for performance and are associated with frequent injury: the DiDimCha, which mimics the Jeong or Pal as a Chinese character and requires movement forward on the heels; the jump on one foot, and the YeonPungDae, a dance step in which dancers turn round and round as if flying through the sky, with repetitive flexion and extension at the waist.

In kinetic analysis, the superior-inferior vector of the ground reaction force (GRF) provides an index of the up-and-down power generated by dancers and the resultant effect on the body. Therefore, the aim of our study was to identify the difference in the magnitude of the superior-inferior GRF according to injury area and the three representative movements of Korean traditional dance, namely the DiDimCha, the jump on one foot and the YeonPungDae.

Participants were 18 female dancers, classified into three groups based on their history of lower limb injury: the knee injury group (n=5; age, 19.86±0.90 yr; height, 164.79±3.69 cm; weight, 53.50±3.51 kg; dance experience, 100.57±52.79 months); the ankle injury group (n=5; age, 19.43±0.79 yr; height, 165.50±4.51 cm; weight, 50.67±5.86 kg; dance experience, 91.14±48.61 months); and the no injury group (n=8; age, 21.00±1.05 yr; height 166.60±4.35 cm; weight, 49.62±2.13 kg; dance experience, 132.60±39.84 months).

The GRF was subdivided into three events for analysis, defined as follows: event 1, touch-down of the left foot on the force platform; event 2, body support on the left foot; and event 3, the moment immediately preceding the left foot coming off the force platform. All three events are included in the DiDimCha, jump on one foot and YeonPungDa. All GRF were recorded using a 9260AA6 Kistler force platform (Winterthur, Switzerland).

The mean ± standard deviation was calculated for the GRFs for each movement, with between-group differences analyzed using a one-way analysis of variance. Statistical analyzes were performed using SPSS (version 18.0; Chicago, IL, USA). Statistical significance was set at *P*<0.05.

The superior-inferior GRF for the three-movement types is summarized in Table 1. There were no between-group differences in the magnitude of the superior-inferior GRF across each of the three events (*P*>0.05).

**Table 1:** Magnitudes of the superior-inferior ground reaction force for the three common movement patterns in Korean dance: DiDimCha, jump on one foot, and the YeonPungDae

**Korean traditional dance movements**	**Group**	**Superior-inferior ground reaction force (N)**	**F**	**P**
DiDimCha	Knee injury	6.67 ± 1.32	0.635	0.544
Event 1	Ankle injury	6.23 ± 1.24		
	No injury	7.08 ± 1.40		
DiDimCha	Knee injury	599.00 ± 40.71	2.106	0.156
Event 2	Ankle injury	552.44 ± 46.25		
	No injury	566.56 ± 27.87		
DiDimCha	Knee injury	5.83 ± 0.69	1.585	0.237
Event 3	Ankle injury	9.32 ± 7.42		
	No injury	5.63 ± 0.48		
Jump on one foot	Knee injury	18.17 ± 16..00	0.892	0.431
Event 1	Ankle injury	41.53 ± 54.19		
	No injury	21.82 ± 12.59		
Jump on one foot	Knee injury	1136.92 ± 293.38	1.169	0.337
Event 2	Ankle injury	988.91 ± 148.58		
	No injury	1168.49 ± 183.87		
Jump on one foot	Knee injury	5.88 ± 0.59	2.748	0.096
Event 3	Ankle injury	7.56 ± 3.03		
	No injury	5.46 ± 0.28		
YeonPungDae	Knee injury	7.63 ± 3.08	0.977	0.399
Event 1	Ankle injury	12.00 ± 5.90		
	No injury	9.31 ± 5.32		
YeonPungDae	Knee injury	75.42 ± 33.73	0.603	0.560
Event 2	Ankle injury	160.96 ± 71.98		
	No injury	110.63 ± 39.11		
YeonPungDae	Knee injury	6.79 ± 0.95	1.559	0.243
Event 3	Ankle injury	9.52 ± 3.78		
	No injury	5.19 ± 5.56		

Data are presented as mean ± standard deviation

Knee injury, 5 dancers; ankle injury, 5 dancers; no injury, 8 dancers.

Event 1, contact of the left foot with the force platform; event 2, body support on the left foot; and event 3, the moment just preceding the left foot coming off the force platform. //*P*-value, evaluated using a one-way analysis of variance

The knee, ankle, and no injury groups could not be differentiated by the superior-inferior GRF across the three basic movements of Korean traditional dance. Therefore, other factors may explain differences in injury pattern, including differences in diet, the relative strength and power of the lower limbs, and technical skill in absorbing the GRF. Future studies, including larger sample sizes, a more diverse injury group, and stringent control are needed to identify risk factors for injury in Korean traditional dance.

## References

[1] Jang HR Lee JH Lee SK (2014). The comparison of body composition, functional performance ability, talar tilt angle and foot injuries in accordance with the functional ankle instability in high school traditional Korean dance majors. The Korea Dance Education Society Journal, 25(2):149–162. (In Korean)

[2] Kadel N (2014). Foot and ankle problems in dancers. Phys Med Rehabil Clin N Am, 25(4):829–844. 25442161 10.1016/j.pmr.2014.06.003

[3] Smith PJ Gerrie BJ Varner KE (2015). Incidence and Prevalence of Musculoskeletal Injury in Ballet: A Systematic Review. Orthop J Sports Med, 3(7):2325967115592621. 10.1177/2325967115592621 PMC4622328 26673541

